# Main Trend Extraction Based on Irregular Sampling Estimation and Its Application in Storage Volume of Internet Data Center

**DOI:** 10.1155/2016/9328062

**Published:** 2016-12-20

**Authors:** Beibei Miao, Chao Dou, Xuebo Jin

**Affiliations:** ^1^Baidu, Inc., Beijing 100085, China; ^2^Center of Quality Engineering, AVIC China Aero-Polytechnology Establishment, Beijing 100028, China; ^3^School of Computer and Information Engineering, Beijing Technology and Business University, Beijing 100048, China

## Abstract

The storage volume of internet data center is one of the classical time series. It is very valuable to predict the storage volume of a data center for the business value. However, the storage volume series from a data center is always “dirty,” which contains the noise, missing data, and outliers, so it is necessary to extract the main trend of storage volume series for the future prediction processing. In this paper, we propose an irregular sampling estimation method to extract the main trend of the time series, in which the Kalman filter is used to remove the “dirty” data; then the cubic spline interpolation and average method are used to reconstruct the main trend. The developed method is applied in the storage volume series of internet data center. The experiment results show that the developed method can estimate the main trend of storage volume series accurately and make great contribution to predict the future volume value. * *

## 1. Introduction

In general, the internet data center stores a huge scale of data, for example, the data of search engines and the data of E-commerce services. It is necessary to predict the future storage volume value of a data center, because it is helpful for operation engineers to make purchasing devices plan for data center. As the devices always have limited warranty time and the maintenance cost is large, it is better to buy devices when needed, because this will cost less. Meanwhile, considering the devices transportation delay, engineers have to buy devices * *in advance to offer enough storage space for the increasing data. Thus, making an accurate prediction result for storage volume series is very important. However, the collected storage data often contains white noise, outliers, and missing data (replaced by 0) besides the real main trend. Such “dirty” data adds great difficulties to make accurate prediction [1]. In fact, dirty data will inflict daunting waste, which had cost US businesses 600 billion dollars each year [2]. Thus, it is very important to clean the dirty data and extract its main trend.

**Figure 1:**
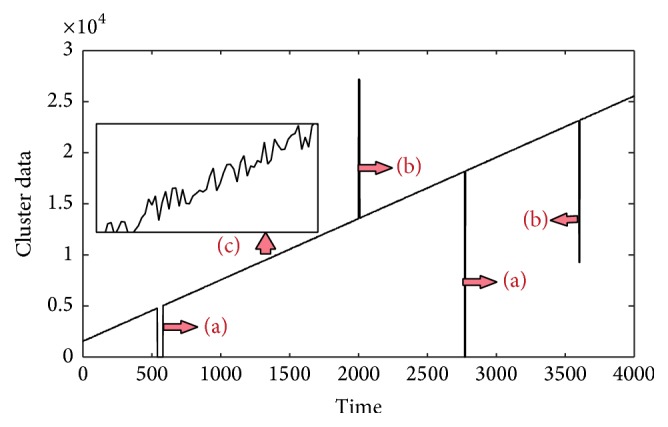
The dirty storage volume time series.

**Figure 2:**
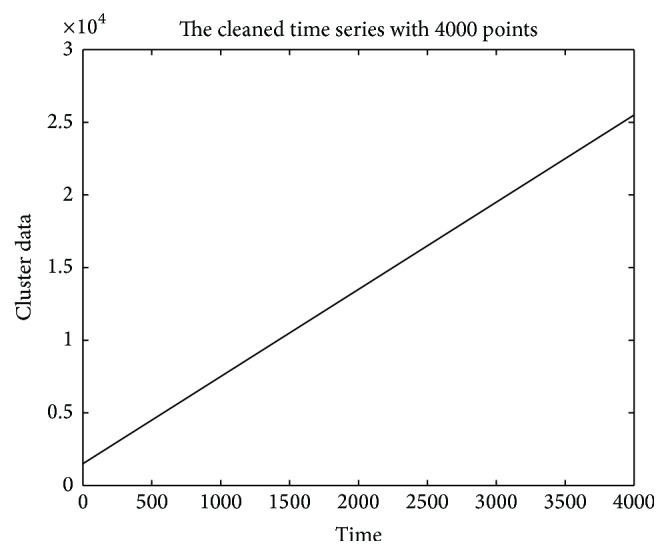
The main trend of storage volume time series.

Figure 1 shows a storage volume series of a data center, which comes from an internet company (the values have been desensitized). Figure 1 labels the main features of the dirty time series data. Feature “a” in Figure 1 represents the missing data, which may be caused by machines' sudden halt or data collection subsystem. Feature “b” represents the outliers, which may be the wrong record data caused by “bugs” in the record programme. Feature “c” represents the noise which is the change of the usage and the enlarged subplot shows the detail fluctuation of the noise in the main trend.  Figure 2 gives the main trend of Figure 1, which * *is the trend we expect to be used for the further prediction of the storage volume. Therefore, our assignment is to extract the main trend in Figure 2 from the “dirty” series in Figure 1.

In fact, there are many techniques of extracting the main trend. For example, [3] employs an STL method to decompose the main trend based on Loess, while [4] applies a piecewise approximation method to extract the underlying long-term trend. Reference [5] explores a quantile regression method to extract the main trend. Though these methods can achieve the main trend, they still have some shortcomings. There are too many parameters and decomposition steps in STL method. Therefore, it is difficult to initialize the parameters and the computation costs are very large. The piecewise approximation method [4] can ignore the distortion of outliers and have good extraction result for the long-term trend. But it cannot give specific data of the main trend because it estimates the long-term trend by median values in certain window. As for the regression method [5], it cannot obtain the main trend directly and has to go further by using interpolation methods to get the main trend, for example, the linear or B-spline interpolation method. This may cause overfitting when the time series is short or achieve poor extraction result because the large blocks of outliers would distort the spline and yield a large number of incorrect interpolation results.

Unlike the mentioned approach, this paper offers an estimation method to achieve the main trend. To the best of our knowledge, the Kalman filter can estimate the dynamic features of time series, as well as its main trend. What is more, researchers have received a lot of results about Kalman filter [6–9], by which the extracted high dimension characters can also be used to predict the tendency of series [10–12] or make further decision through other methods like the intelligence method [13, 14]. For example, [6] gives a real-time correction method to estimate data online and forecast the water stage, which is effective and fast. Reference [15] considers a prediction problem for multivariate time series and proposed an online model by echo state network (ESN) based on square root cubature Kalman filter.

However, the above Kalman filter methods are based on regular sampling estimation, which means that all of the measurements will be used. If we employ these methods on the storage volume time series directly, the outliers and missing data will greatly degrade the estimation performance. For our “dirty” time series case, we prefer to use some of the measurement data so as to discard the outliers and the missing data. Thus, the “irregular” estimation method will be helpful.

As for the irregular estimation methods, researchers have got lots of results; for example, [16] develops a method to handle time-varying and uncertain delay problem. Based on the modification of the Kalman filter and the negative-time measurement update strategy, [17] used the full augmented order models to handle the long delay problem for the networked control systems and scarce measurement problem for out-of-sequence measurements. Reference [18] discussed the irregular estimation method in detail and transformed the irregular sampling time to a time-varying parameter by calculating the matrix exponential with inverse Laplace transform method. Based on the statistic relation between autocorrelation function and the covariance of Markov random processing, [19] develops a model to track video signal by Kalman filter, which can adaptively adjust the model parameters while tracking and obtain good estimation performance even at a very low irregular rate. These research results show us that the Kalman filter based on irregular sampling estimation can use the part of the measurement data and we hope it will help us to cut down the effect of the outliers and the missing data.

The result of irregular sampling estimation is the compressed data series with unknown amount of series and sampling intervals, which will confuse the following prediction of the future main trend. So we apply cubic spline method to interpolate the storage volume time series and reconstruct the whole time series with the same number of the former series. Also, we note that the irregular estimation may select some “dirty” measurements. Such data may distort the estimation, which will cause the quality of the main trend with poor performance. To get robust main trend, we estimate and reconstruct the time series several times instead of only once. Then, an average method is used to achieve the main trend. Part of the developed method has been mentioned in the conference paper [20] with 4 pages. By comparison, this manuscript gives the details of the main trend extraction method and discusses the experiments more comprehensively.

This paper is organized as follows.  Section 2 details the main trend estimation algorithm, including the irregular sampling estimation based on Kalman filter, the cubic spline interpolation reconstruction method, and the averaging method.  Section 3 gives the experiment results. The developed method is applied for the storage volume of the internet data center; meanwhile the results of some reference methods, such as the Piecewise Median Underlying (PMU) method, the local regression smoothing (Loess) method, and the Moving Average (MA) smoothing method, are also given. Conclusions and future works are presented in Section 4.

## 2. Main Trend Estimation Algorithm

Before introducing the algorithm, we have to give the definition of Compression Sampling Rate (CSR) to value the degree of compression:

(1) $$\begin{array}{c} CSR=\frac{N_{s}}{N}, \end{array}$$

where *N*_*s*_ is the number of selected data and *N* is the total number of original storage volume time series. We can note that lower CSR means higher compression degree. By using the irregular sampling method, we can retain the main information of original storage volume time series under a low CSR value.

**Figure 3:**
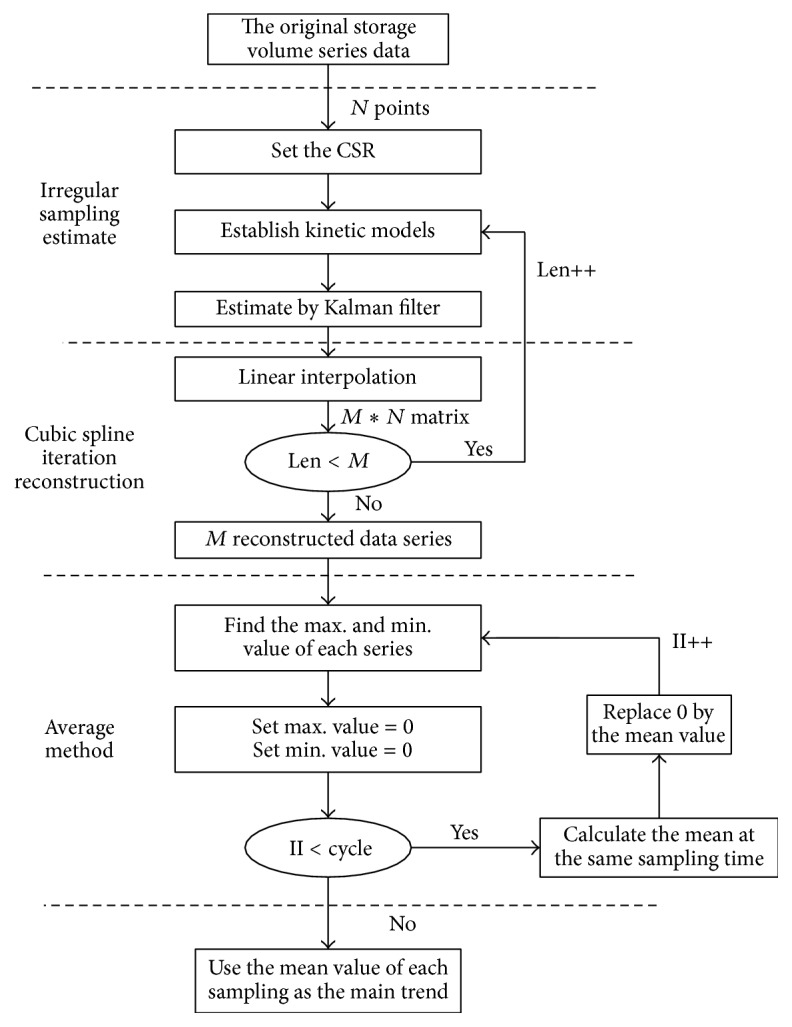
The flow chart of main trend estimation algorithm.

Figure 3 gives the flow chart of the main trend estimation algorithm. The main trend estimation algorithm contains three parts: the compressed estimation step by Kalman filter, the cubic spline interpolation reconstruction method, and the average method. By using the irregular sampling method, we can compress the original dirty data series and try to discard outliers and missing data. The interpolation method is helpful to achieve reconstructed time series with the same length as the original storage volume time series. To avoid the influence of some “lucky” selected outliers, we estimate the time series *M* times and obtain *M* time series with the same CSR value. The average method is used to obtain final extracted main trend.

### 2.1. The Irregular Sampling Compression and Kalman Filter Estimation Method

Our research is designed for discrete time series, and the following is the Kalman filter equations:

Initialization: *k* = 0
  (2) x^0 ∣ 0=V0,P0 ∣ 0=P0,α0=α0,δw20=δw02,λ=λ0
Recursion: *k*≔*k* + *δ*

Here *δ* represents the interval between two pieces of input data. Usually *δ* > 1, so we can compress the series and use the Kalman filter to extract the basic trend. The method about how to select *δ* is discussed in Note 1.

*(a) Prediction*

(3) x^k+δ ∣ k=Φk+δ,kx^k ∣ k,Pk+δ ∣ k=Φk+δ,kPk ∣ kΦTk+δ,k+Qk.

*(b) Update*

(4) x^k+δ ∣ k+δ=x^k+δ ∣ k+Kk+δyK+δ−Hx^k+δ ∣ k,Kk+δ=Pk+δ ∣ kHTHPk+δ ∣ kHT+RT,Pk+δ ∣ k+δ=I−Kk+δHPk+δ ∣ k,

where the observation vector *y*(*k*) is the storage capacity time series with *N* points and *k* = 1,2, 3,…, *N*;  $\hat{x}(k+\delta ,k+\delta )$ is the estimated current time series data. *I* is a unit matrix and *R* is the covariance of the series noise. *ϕ*(*k* + *δ*, *k*) is the process transformation matrix, *Q*(*k*) is the process noise covariance matrix, and *H* is the observation transformation matrix.

From (2)–(4), we can see that the system parameters of *ϕ*(*k* + *δ*, *k*), *Q*(*k*), *H*, and *R* are important to the Kalman filter, in which *ϕ*(*k* + *δ*, *k*) and *Q*(*k*) are called the process models and *H* and *R* are called measurement models. To capture the dynamic characters, researchers have given many models for estimation. Notes 2 and 3 will give more specific information about dynamic models.

**Note 1 (the selection about *δ*).**

The initial *δ* is set as 0 and assume Sa is a uniform distribution random vector with *N* dimension, where Sa(*i*)∈(0,1), *i* = 1,2,…, *N*. Then we introduce a constant named* A*, where *A* ∈ (0,1) corresponding to CSR. For example, *A* is 0.7 means the CSR value is (1−0.7) *∗* 100% = 30%.

**Figure 4:**
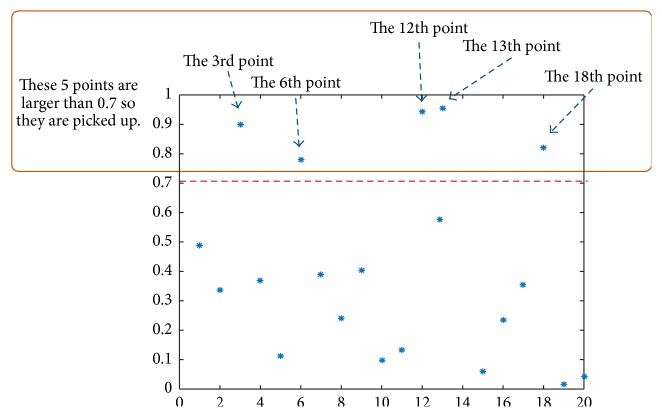
The relation of *A* and *δ*.

**Figure 5:**
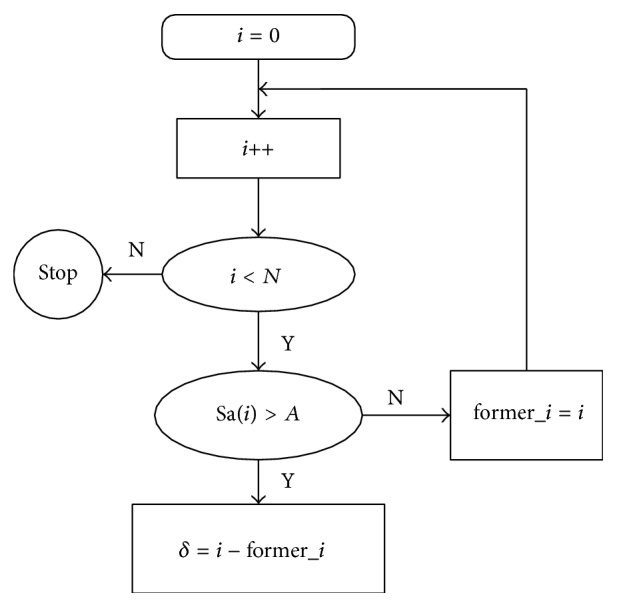
The flow chart of obtaining *δ*.

We obtain the interval value by comparing Sa(*i*) and *A*. Only the *i*th data with Sa(*i*) > *A* is picked up and *δ* is calculated by two adjacent picked pieces of data. We give an example for the relation of *A* and *δ* and how to calculate *δ* is shown in Figure 4. In Figure 4, *A* is set as 0.7, and 20 points Sa(*i*)∈(0,1), *i* = 1,2,…, 20, are created by a uniform distribution random vector. We can see that only the 3rd, 6th, 12th, 13th, and 18th are larger than 0.7; therefore we can get *δ* = 3, 3, 6, 1, and 5. The flow chart is shown in Figure 5.

**Note 2 (the selection of process models).**

The process model described the changing relations about the main trend. Some inertia model had been developed by the researchers, such as constant-velocity (CV) model, constant-acceleration (CA) model, Singer model, the “current” model, and the adaptive model. CV [21] assumes that the acceleration is a Wiener process or, more generally and precisely, the acceleration is a process with independent increments, which is not necessarily a Wiener process. It is simply referred to as CA or more precisely “nearly-constant-acceleration model” [22]. The Singer model in [23] assumes the acceleration as a first-order semi-Markov process with zero mean, which in essence is a priori model since it does not use online information about the target maneuver, and it can be made adaptive through some parameters.

An acceleration model, called the “current” model [23], is in essence a Singer model with an adaptive mean, that is, a Singer model modified to have a nonzero mean of the acceleration. The “current” model can use the online information and replace the a priori (unconditional) probability density of acceleration in Singer model by a conditional density, that is, Rayleigh density. Clearly, this conditional density carries more accurate information than a priori density.

The above models all need prior hypothesis. Based on the statistical relation between the autocorrelation function and the covariance of Markov random processing, [24] develops a model which can adaptively adjust system parameter to dynamics characters of time series online, but this process is complex to compute. In practice, we should choose the appropriate system parameters to suit the data dynamics characters. In our experiments, we use several models for the developed algorithm and discuss the estimation performance.

**Note 3 (the selection of measurement models).**

We give the measurement model for the main trend of the series data as

(5) $$\begin{array}{c} y\left(\;k\;\right)=H\left(\;k\;\right)x\left(\;k\;\right)+v\left(\;k\;\right), \end{array}$$

where *x*(*k*) is the main trend to be extracted. The extraction matrix *H*(*k*) can be set as $H\left(k\right)=\left[\begin{array}{ccc} 1 & 0 & 0 \end{array}\right]$ if the state is defined as a three-dimensional vector. The covariance of the extraction noise *v*(*k*), which we denote as* R*, can be decided by the difference between the main trend and the original data as

(6) $$\begin{array}{c} R=\frac{\sum_{k=k_{0}}^{N_{t}+k_{0}}{\left[y\left(k\right)-x\left(k\right)\right]}^{2}}{N_{t}}, \end{array}$$

where *y*(*k*) is the original data, *x*(*k*) is the expected main trend, *k*_0_ is the positive constant, *k*_0_ ∈ (0, *N*), and *N*_*t*_ is the number of the series to be estimated; *N*_*t*_ ≤ *N* − *k*_0_.

**Figure 6:**
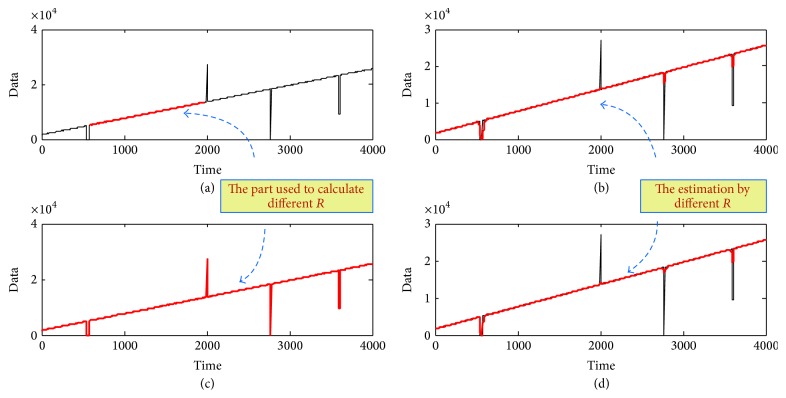
The main trend of storage volume time series.

**Figure 7:**
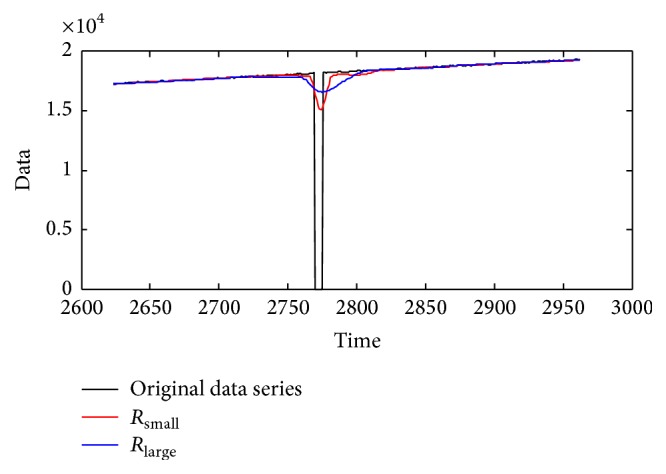
The comparison of different *R* from 2600th to 3000th samples.

To better illustrate the influence of *R*, we choose a segment of the dirty data in Figure 1 with *N*_*t*_ samples to calculate *R* (named as *R*_small_), where *N*_*t*_ ≤ *N* (in the first subplot in Figure 6, the red part is the data selected to be estimated). We also choose the whole dirty time series to calculate *R* (named as *R*_large_), shown in the third subplot in Figure 6, with the “red” part showing the data for estimating. Because of the influence of outliers, *R*_small_ is smaller than *R*_large_. In the second and the fourth subplots in Figure 6, the red lines show the estimation results by *R*_small_ and *R*_large_, respectively. To be more clear, Figure 7 gives an enlarged subplot about the estimated main trend with *R*_small_ and *R*_large_ from 2600th to 3000th samples. From Figure 7, we can conclude that *R*_small_ can get the less estimated point with errors than *R*_large_ and such peak value can be removed by the following average step. Therefore, in practice, *N*_*t*_ is chosen as *N*/3 so as to remove some of the “dirty” data and receive the better results.

As to the expected main trend *x*(*k*), we should choose it based on the practical applications. In general, we know what the main trend should be in practice, as we have mentioned that the “dirty” data of a data center shown in Figure 1 should have the main trend as in Figure 2, where the data engineers give this suggestion based on the knowledge about the data center.

### 2.2. The Cubic Spline Interpolation Reconstruction Method

By the compression and estimation method in Section 2.1, we can obtain the useful information of main trend based on part of the original storage volume series. But the compressed series cannot be used for the further prediction directly because its sampling is irregular. Thanks to *δ* parameter, the specific intervals between two pieces of input data are retained and can be used to reconstruct the whole time series.

The polynomial of cubic spline interpolation *S*(*x*) is a piecewise function of *x*_0_, *x*_1_,…, *x*_*n*−1_, *x*_*n*_ data.Each cubic polynomial value determines the parameters at each mini zone [*x*_*i*−1_, *x*_*i*_] and the node *x*_*i*_ satisfies *a* = *x*_0_ < *x*_1_ < ⋯<*x*_*n*−1_ < *x*_*n*_ = *b*. We have

(7) Sx=16hixi−x3λi−1+x−xi−13λi+yi−1−hi26λi−1xi−xhi+yi−hi26λix−xi−1hi,

where *x* ∈ [*x*_*i*−1_, *x*_*i*_], *i* = 1,2,…, *N* · *CSR*.

Define

(8) μihihi+hi+1,λihi+1hi+hi+1=1−μi,di6hi+hi+1yi+1−yihi+1−yi−yi+1hi=6fxi−1,xi,xi+1.

Equation (8) satisfies the following *n* − 1 equations:

(9) $$\begin{array}{c} \mu_{i}\lambda_{i-1}+2\lambda_{i}+\lambda_{i}\lambda_{i+1}=d_{i},\;i=1,2,\ldots ,n-1. \end{array}$$

Equation (7) has *n* + 1 unknown quantity; if we set *λ*_0_ = *λ*_*n*_ = 0, the value of *λ*_*i*_ (1 ≤ *i* ≤ *n* − 1) and trend(*x*) can be obtained. In our reconstruction, we get cubic spline interpolation values in each [*x*_*i*−1_, *x*_*i*_], where *x*_*i*−1_ and *x*_*i*_ are the adjacent two sampled input storage time series.

### 2.3. The Averaging Method

As mentioned previously, we select the part of the original dirty storage volume series to estimate. Under the assumption that the outliers are little, we can guarantee that most of the selected data can contribute to the main trend data and most of useful information can be extracted when outliers can be discarded and missing data can be handled. However, some of the outliers or the missing data of the real storage volume time series may be “lucky” enough and be selected. In fact, the “lucky” selected data will distort the main trend in the interpolation reconstruction step and decrease the extraction performance. Decreasing the CSR value is surely a way to reduce the possibility of “lucky” one, but too low CSR value may cause some useful information to be lost. So we further use the average method to achieve the main trend after reconstruction step.

The averaging method can be detailed as follows. At first, we use the irregular estimation method *M* times to get *M* main trend series. As to the reconstruction results, the selected “lucky” outliers or missing data are always the max. or min. value among *M* reconstructed series with the same column. Thus, the average method is used to calculate the mean value by discarding the max. and min. value. Repeat several cycle times until the max. and min. are similar to the mean. For simplicity, instead of numerical comparisons, we use the number of cycles to control the end of the cycle. The detailed algorithm is as follows:

(1) Calculate the max. and min. values of each column in *M* × *N* reconstructed time series matrix, where *N* is the number of the time series.
(2) Set the max. and min. value of the row by zeros.
(3) Calculate the mean value of each column in the replaced *M* × (*N* − 2) matrix.
(4) Use the calculated mean value replacing the “zeros.”
(5) Repeat the above steps* II* cycle times, where* II* is a positive constant.

## 3. Experiments and Discussion

In this section, two parameters are used to measure the performance of the developed main trend estimation method: Covariance (Cov) is introduced to evaluate the quality of the estimated main trend and Time of Programming (TP) is applied to measure the calculation cost of different dynamic models:

(10) y^k−yk=ek,Cov=∑k=1NekN,

where *y*(*k*) represents the expected main trend, while $\hat{y}(k)$ represents the estimated main trend by developed algorithm. *N* is the total number of the original storage volume time series.

Section 3.1 gives several reference methods, such as the Piecewise Median Underlying method, the Local regression smoothing (Loess) method, and the Moving Average (MA) smoothing method, whose results have been discussed.  Section 3.2 discussed the developed method of performance based on different models, including CV, CA, Singer, current model, and adaptive model.

### 3.1. Several Reference Approaches for Extracting Main Trend

**Figure 8:**
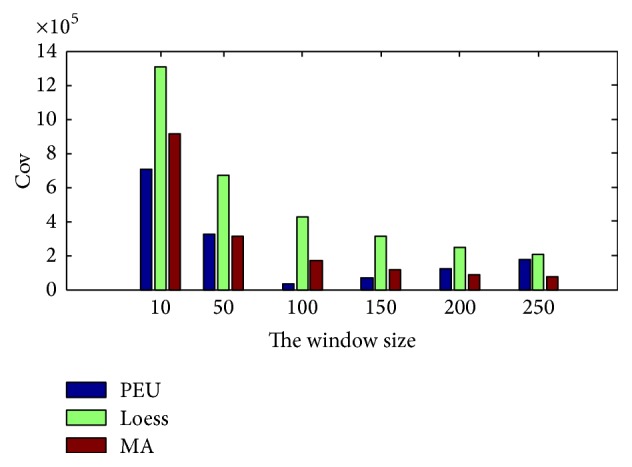
The extraction result by PMU, Loess, and MA.

In this section, the Piecewise Median Underlying (PMU) method [5], the Local regression (Loess) smoothing method [3], and the Moving Average (MA) smoothing method [4] are used for extracting the main trend of the storage volume.  Table 1 gives the Cov result based on the three methods under different window size and Figure 8 shows the extraction result of the three methods.

**Figure 9:**
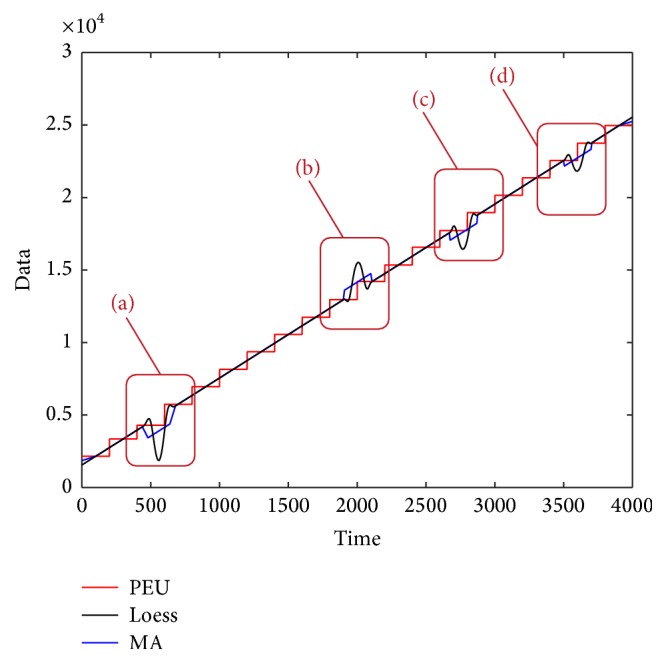
The extracted main trend by PEU, Loess, and MA methods.

**Figure 10:**
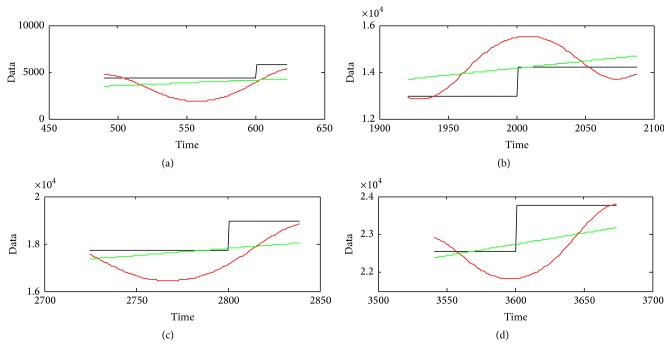
The detailed extraction result by PMU, Loess, and MA methods.

From Table 1 and Figure 8, we can see that the window size can influence greatly the result of main trend extraction by PMU, Loess, and MA methods. For PMU method, there exists a tradeoff between the window size and the extraction performance. In fact, small window size means a better approximation of the expected main trend, while large window size means a more poor approximation result. So the Cov value should be larger along with larger window size. But the existence of missing data and outliers make Cov value very large when the window size is small. That is the reason why a tradeoff (323010) exists in PMU extraction method. For Loess and MA methods, it is easy to find that larger window size means better extraction result. But when the window size is too large, the time delay problem is very obvious. Thus we have to find a tradeoff for these two methods too, for example, the window size with 200.  Figure 9 gives the main trend extraction result of the three methods under their tradeoff. And Figure 10 gives the detailed information of the four windows in Figure 9.

From Figures 9 and 10, we can see that the Loess and MA methods can remove white noise very well, but they cannot deal with the outliers and missing data as well as PMU. Though the PMU has a low Cov value, it depends on the values of the calculating window. What is more, there exists a long lag effect for its trend if the last few points are less than the window.

### 3.2. Extract Main Trend Based on Different System Models Based on Different System Models

**Figure 11:**
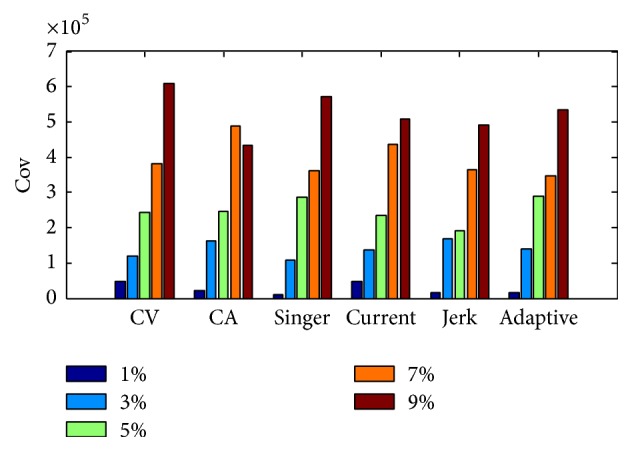
The estimation Cov result of different models.

**Figure 12:**
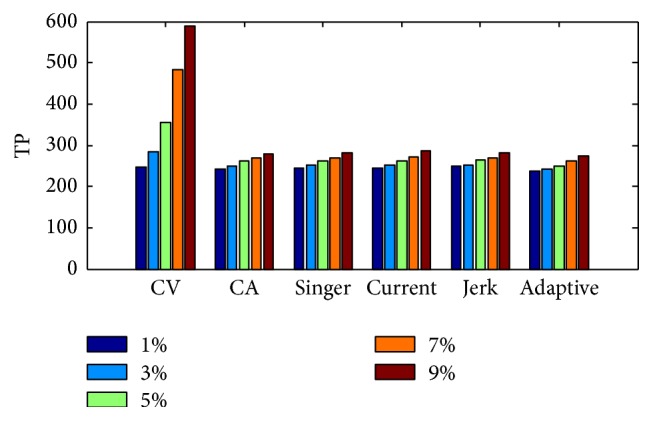
The estimation TP result of different models.

In this section, we will use different system model for the developed extracting method to compare with PEU, Loess, and MA methods; meanwhile we will analyze the performance of developed method based on different system models.  Table 2 gives the extraction Cov with the different system dynamic models and CSR. Figures 11 and 12, respectively, give the Cov and TP values about different dynamic models and different CSR values. Comparing Tables 1 and 2, we can conclude that our developed method is better than PMU, Loess, and Moving Average smoothing methods, because the Cov value is smaller, especially when the CSR value is small. We admit that the computation cost of the developed method is larger than other three methods, but the computing time cost will still be within the limitation of practical application.

From Table 2, we can see that smaller CSR means better main trend estimation result when the dynamic model is determined. It is about half time down of Cov values when the CSR is 2% smaller. The reason is that small CSR can reduce the probability of selecting “lucky” dirty data.

**Figure 13:**
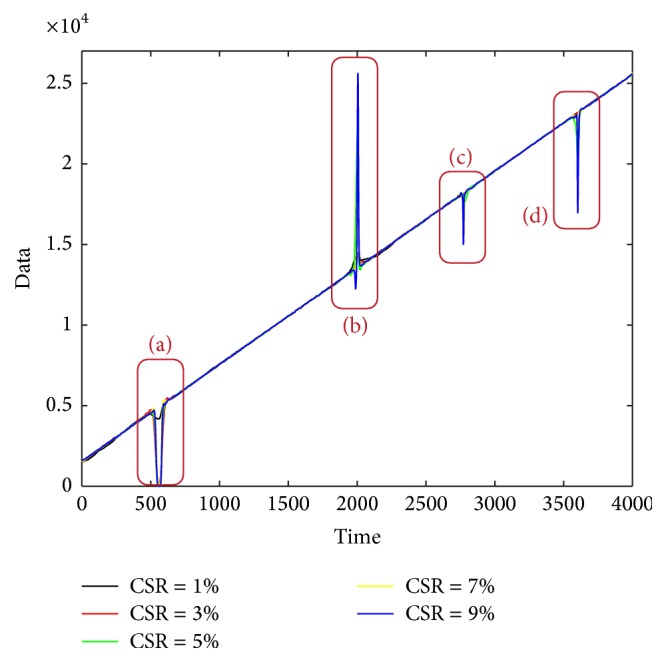
The estimation result by Singer model under different CSR.

**Figure 14:**
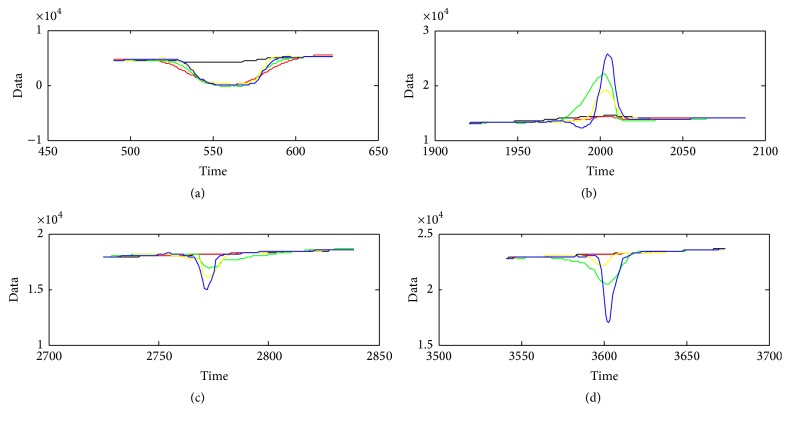
The enlarged details of Figure 13.

Figure 13 gives the estimated trends with the Singer model for different CSR values, and Figure 14 gives the enlarged details of the windows in Figure 13. From the figures, we can note that lower CSR value is helpful to achieve better main trend.

## 4. Conclusions

**Figure 15:**
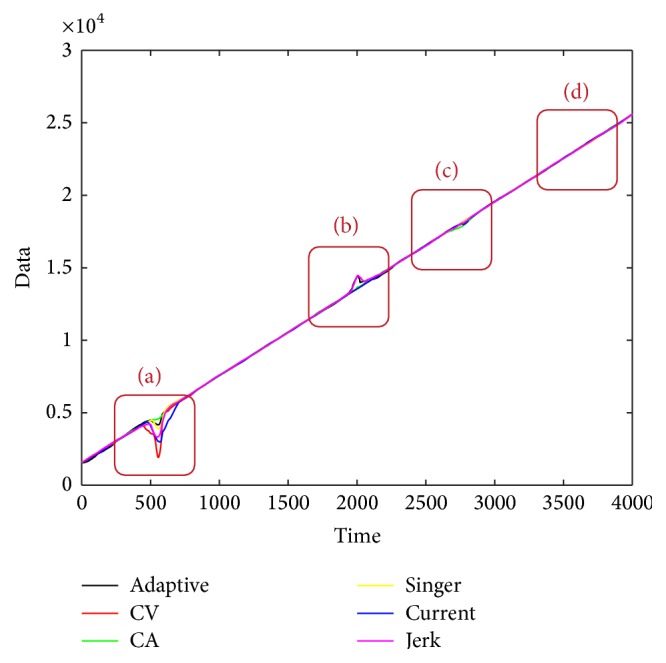


**Figure 16:**
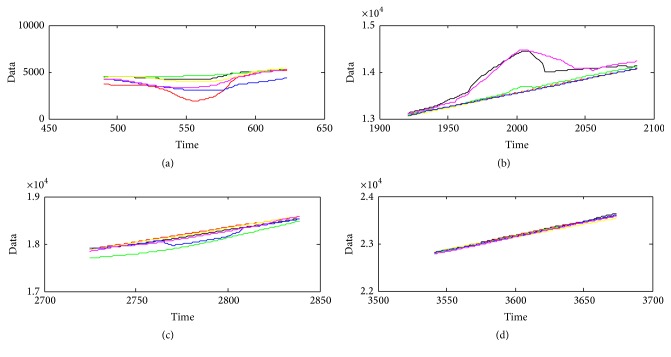


The estimation result may be different by different dynamic models with the set CSR value.  Figure 15 gives the estimation result about different models when CSR is 1%. And Figure 16 gives the detailed estimation information where there exists large block of outliers. By the different estimation covariance in Table 2, it can be concluded that different model can achieve different estimation results. On the other hand, less CSR means less TP value, which means less computing time cost. We also note that less CSR can also result in less Cov values of the estimation. This excellent characteristic helps in choosing an appropriate CSR for good estimation when the Cov and TP values are small. From Table 2, we can conclude that the developed method can achieve the best main trend when CSR is 1% with the Singer model, because it can achieve the lowest Cov value with the lowest TP.

This paper gives a method to estimate the main trend for the “dirty” time series of the storage volume series of the data center. We combine the irregular compressions based on Kalman estimation method together as well as the cubic spline interpolation reconstruction algorithm to extract the useful main information and then the average method is used to get the final main trend. We test this developed method on a storage volume series offered by an Internet company. It can be found that our developed method can estimate the main trend of a storage volume time series more accurately than PMU, Loess, and MA methods. And the accurate main trend is helpful for predicting the future storage volume value. We would like to mention that the developed algorithm has been used in practice and, together with the prediction algorithms, it has received high accuracy in practice.

## Figures and Tables

**Table 1:** The Cov of main trend extraction result based on PMU, Loess, and MA.

Cov	PMU	Loess	MA
10	704090	1307400	915220
50	323010	672790	309860
100	**32780 **	427980	167570
150	71339	314470	114510
200	124360	**244900 **	**85857 **
250	174170	206780	71517

**Table 2:** The Cov/TP main trend estimation result.

CSR	CV	CA	Singer	Current	Jerk	Adaptive
1%	46214/246.60	21834/243.11	**11278/244.60**	47011/245.23	15680/248.69	14839/237.18
3%	119460/285.06	163920/250.55	107880/252.01	137070/251.68	168470/252.27	140280/242.28
5%	243250/355.91	247660/261.56	287760/261.34	233420/261.83	192730/264.86	288430/250.64
7%	382880/483.28	488200/268.93	360140/269.89	436360/272.67	365600/270.34	348420/260.81
9%	610100/588.35	433290/279.60	570830/281.79	507100/287.21	492320/280.91	534560/273.98
